# ADOS-Eye-Tracking: The Archimedean Point of View and Its Absence in Autism Spectrum Conditions

**DOI:** 10.3389/fpsyg.2021.584537

**Published:** 2021-03-18

**Authors:** Ulrich Max Schaller, Monica Biscaldi, Anna Burkhardt, Christian Fleischhaker, Michael Herbert, Anna Isringhausen, Ludger Tebartz van Elst, Reinhold Rauh

**Affiliations:** ^1^Department of Child and Adolescent Psychiatry, Psychotherapy, and Psychosomatics, Medical Center – University of Freiburg, Faculty of Medicine, University of Freiburg, Freiburg, Germany; ^2^Department of Psychiatry and Psychotherapy, Medical Center – University of Freiburg, Faculty of Medicine, University of Freiburg, Freiburg, Germany

**Keywords:** autism spectrum disorder, eye-tracking, autism diagnostic observation schedule, social cognition, social schemas, online social cognition, face perception, social interaction

## Abstract

Face perception and emotion categorization are widely investigated under laboratory conditions that are devoid of real social interaction. Using mobile eye-tracking glasses in a standardized diagnostic setting while applying the Autism Diagnostic Observation Schedule (ADOS-2), we had the opportunity to record gaze behavior of children and adolescents with and without Autism Spectrum Conditions (ASCs) during social interaction. The objective was to investigate differences in eye-gaze behavior between three groups of children and adolescents either (1) with ASC or (2) with unconfirmed diagnosis of ASC or (3) with neurotypical development (NTD) during social interaction with an adult interviewer in a diagnostic standard situation using the ADOS-2. In a case control study, we used mobile eye-tracking glasses in an ecologically valid and highly standardized diagnostic interview to investigate suspected cases of ASC. After completion of the ASC diagnostic gold standard including the ADOS-2, the participants were assigned to two groups based on their diagnosis (ASC vs. non-ASC) and compared with a matched group of neurotypically developed controls. The primary outcome measure is the percentage of total dwell times assessed for different areas of interest (AOI) with regard to the face and body of a diagnostic interviewer and the surrounding space. Overall, 65 children and adolescents within an age range of 8.3–17.9 years were included in the study. The data revealed significant group differences, especially in the central-face area. Previous investigations under laboratory conditions gave preferential attention to the eye region during face perception to describe differences between ASC and NTD. In this study – using an ecologically valid setting within a standard diagnostic procedure – the results indicate that neurotypically developed controls seem to process faces and facial expressions in a holistic manner originating from the central-face region. Conversely, participants on the Autism Spectrum (tAS) seem to avoid the central-face region and show unsystematic gaze behavior, not using the preferred landing position in the central-face region as the Archimedean point of face perception. This study uses a new approach, and it will be important to replicate these preliminary findings in future research.

## Introduction

In the scope of social communication, the human face is one of the prime sources for relevant nonverbal information and an effective key instrument, producing information in a dynamic and highly efficient manner.

Within two gaze fixations, we are able to recognize a face (Hsiao and Cottrell, 2008); only a few fixations later, we can draw conclusions about gender, age, identity, ethnicity, attractiveness, health, and particularly about the emotional state of a human counterpart (Jack and Schyns, 2015). Even minute movements of unconscious facial mimicry can affect the process and development of a social interaction (Dalton et al., 2010). It follows that many researchers metaphorically speak of empathy, mimicry, and social gaze as glue for social communication (Lakin et al., 2003; Baron-Cohen and Wheelwright, 2004; Kuzmanovic et al., 2009), and the face can be considered as the focal point of direct social interaction.

People with Autism Spectrum Conditions (ASCs) show a wide range of clinical characteristics, but difficulties in social interaction and nonverbal communication are considered as core challenges for people on the Autism Spectrum (tAS). Several groundbreaking eye-tracking studies have illustrated that individuals on tAS show reduced attention to salient social stimuli, especially in the eye region (Klin et al., 2002; Jones et al., 2008; Jones and Klin, 2013). These studies, however, are all investigations conducted under laboratory conditions in which the stimulus material was detached from the participant and presented *via* screen.

What is lacking in this type of stimulus presentation is the interactive aspect of social communication in the real world (Foulsham, 2020). Pictures, comics, photographs, and video sequences of social content are passive and self-contained; in most cases, the problem definition focuses on a specific task, which the participant has to fulfill as an (passive) observer, not as an (participating, active) interactor. It is, therefore, a form of studying “offline” social cognition (Schilbach, 2014) with high internal validity, but information on the context, functioning, and processing of the rules of “online” social interaction remains poor.

Interpersonal social interaction is distinguished by a permanent exchange of social signs that are simultaneous and time constrained using limited cognitive resources and bounded rationality (Simon, 1956). In addition to the verbally mediated content, one has to perceive and categorize paraverbal modulations, body posture, gesture, and especially facial expressions such that the given response meets the expectations of the counterpart.

In order to reduce the contingency and complexity of such a social situation and to allow for context-adequate communication, the expectations of expectations (Luhmann, 1987) of the interactors have to be coupled with social schemas and scripts (Bartlett, 1932; Schank and Abelson, 1977; Augoustinos and Walker, 1995; Schaller and Rauh, 2017; Schaller et al., 2019).

As such, real-time social interaction with natural human agents in a specific contextual framework places very different demands on participants than a purely observational offline task.

Looking now at available meta-analyses concerning eye-tracking in ASC, it becomes clear that studies of autistic children and adolescents as well as those of adults on tAS show significantly reduced gaze-fixation to the eye-region of faces. A closer look at the methodology of the included studies reveals that all are based on an offline social cognition design, even those that have been specified as interactive. In this context, “interactive” is described as any static or dynamic image involving at least two human or animated figures that are posed in a possible state of interaction that has to be observed by the participant (Papagiannopoulou et al., 2014; Frazier et al., 2017).

Other survey articles, however, make clear that results found by using offline cognition are not consistent according to the hypothesis that individuals on tAS show reduced fixation of the eye-region (Guillon et al., 2014).

In their eye-tracking study, Chevallier et al. (2015) show that the ecological relevance of social stimuli is an important factor to measure social attention and motivation in ASC. Therefore, they use an interactive task. The interaction, however, is that of characters shown in a video and by no means an interaction between social stimulus and participant. On the other hand there is current evidence showing that individuals on tAS spend less time to social stimuli especially in complex social situations with more than one person (Chita-Tegmark, 2016). These examples illustrate the big heterogeneity of the methodological positions in eye-tracking studies concerning social cognition in ASC.

From this, the following question arises, to what extent do the demands of complex social cognition alter gaze behavior if the given task requires an individual to be an interactor in an ecologically valid social situation instead of just a passive observer in a detached offline task.

To answer this research question, we focused on the “Conversation and Reporting” activity within the Autism Diagnostic Observation Schedule, 2nd edition (ADOS-2, Hus and Lord, 2014) and applied a mobile eye tracking system during a 10-min sequence of social interaction in order to assess and compare gaze behavior of people with suspected diagnoses of ASC with neurotypically developed controls.

The hypothesis of this study states that – in an ecologically valid, socially dynamic situation – participants on tAS will show different proportions of total dwell times in areas of interests (AOIs) concerning the face of the interviewer when compared (a) to patients with other psychiatric diagnoses and (b) to neurotypically developed controls.

## Materials And Methods

### Participants

Study participants were recruited from the population of referrals with suspected ASC from the outpatient clinic of the Department of Child and Adolescent Psychiatry, Psychotherapy and Psychosomatics of the Medical Center of the University of Freiburg within the time period from February 2014 to February 2016. In total, there were 290 children and adolescents with initial suspicion of ASC that could be tested with ADOS-2 Module 3 or 4 as part of the gold standard diagnostics for ASC.

Inclusion criteria for participation in the study were the following: age range from 8.0 to less than 18.0 years; IQ ≥ 70; full command of the German language; and parental consent.

The list of exclusion criteria consisted of (i) vision defects that required visual acuity correction devices (>±1.5 dpt; wearing glasses is not possible in combination with the mobile eye-tracking device) and (ii) patients with severe ADHD symptoms, which could not be completely controlled by medication (high risk of invalid mobile eye-tracking recordings). Within the control group, children and adolescents with values in the clinical range for the Social Responsiveness Scale (SRS; total raw score cut-off ≥75; Bölte and Poustka, 2008) and the Child Behavior Checklist (CBCL/4–18; T-score >63 on Internalizing, Externalizing, and Total Scales; Greenbaum et al., 2004) were also excluded from further analyses.

Although only few eye-tracking studies with individuals on tAS report large effect sizes, it was clear from the beginning that we could not achieve a sample size that would have been sufficient to reveal medium effects. In order to be able to detect at least large effects between the clinical samples and the control group (power = 0.80 and alpha = 0.05), power calculations indicated *n* = 20 per sample and hence a total sample size of 60 children (as computed by G*Power, version 3.1.3; Faul et al., 2007).

Information on this study was provided to the parents or caregivers and the children themselves before their voluntary participation *via* a written information letter as well as a verbal description. Prior to a child’s participation, the parent or caregiver was required to sign a written informed consent form.

**Figure 1:**
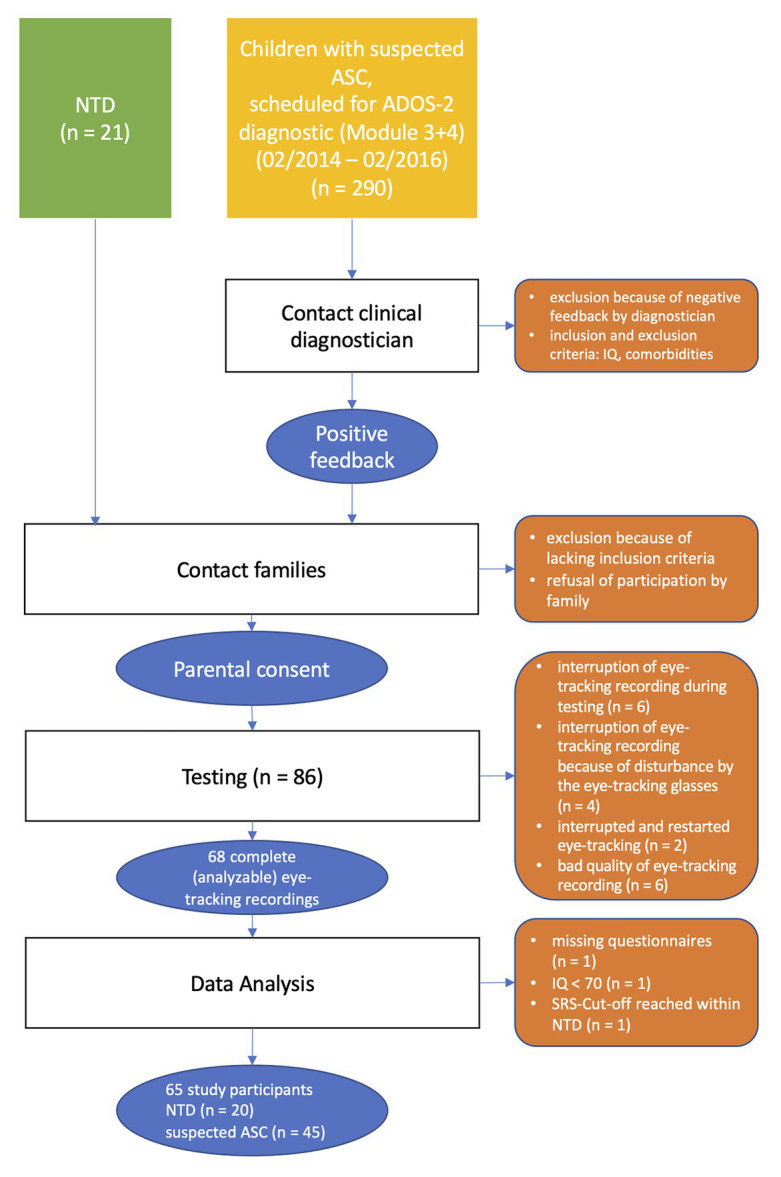
Flow of participants.

Initially, 63 children and adolescents were recruited for the study. Some were later excluded from further analyses (see Figure 1 for the flow of participants).

In the end, the data of 45 participants with suspected ASC could be included in the statistical analysis. In addition, a control group of neurotypically developed children and adolescents (neurotypical development, NTD; *n* = 20) was recruited and matched by age, IQ, and gender. In sum, the total sample consisted of *N* = 65 children and adolescents.

All participants were tested with ADOS-2 Module 3 or 4 (Hus and Lord, 2014), depending on age. It should be noted that none of the participants had been diagnosed with ASC prior to the study.

### Accompanying Instruments

#### Autism Diagnostic Observation Schedule-2

The “Autism Diagnostic Observation Schedule-Generic” (ADOS-2; Hus and Lord, 2014) is a semi-structured, well-validated observational assessment. It consists of five modules: Toddler Module (pre-verbal/single words; 12–30 months old), Module 1 (pre-verbal/single words; 31 months and older), Module 2 (phrase speech), Module 3 (fluent speech; child/adolescent), and Module 4 (fluent speech; adolescent/adult). Each module consists of 11–15 parts called as “activities.” In the present study, only Module 3 and Module 4 were administered; both modules incorporate the activity *Conversation and Reporting*, during which relevant eye-tracking data was registered.

Each ADOS-2 module has its own classification algorithm that is based on three components with two cut-offs each: (1) scale *Communication Total*, (2) scale *Social Interaction Total*, and (3) combined score of *Communication + Social Interaction Total*. According to the attained cut-offs, it defines three classifications: (a) *autism* or (b) *autism spectrum* or (c) *non-spectrum*. One relevant item is *B1*. *Unusual eye contact* judged by the diagnostician across all activities. This item has only two dichotomous values: 0 = *appropriate gaze* or 2 = *purely modulated eye contact*.

Concerning the psychometric properties of the ADOS-2 (or its precursors), there exist many studies since its development in the 1980s. For the German version of ADOS, Bölte and Poustka (2004) report the following information: the interrater and retest reliability were shown both at the level of diagnoses (*κ_w_* = 1.00 and *κ_w_* = 0.62) and at the level of scales (*r* = 0.84 and *r* = 0.79) as good. The internal consistency of the algorithm scale for modules 1–4, with values from *r* = 0.78 to 0.89, was also acceptable to good. The validity/diagnostic convergence with the Autism Diagnostic Interview-Revised (ADI-R) was 79% (*κ* = 0.23).

#### Autism Diagnostic Interview - Revised

The gold standard of ASC diagnostic procedure combines ADOS-2 with the ADI-R (Bölte et al., 2006). The ADI-R consists of a semi-structured caregiver interview. Ninety-three items investigate current ASC-typical behaviors and developmental history. The interview took place in absence of the child and was applied for all 45 participants with suspected ASC. The ADI-R diagnostic algorithm consists of the following subscales: (1) Qualitative Abnormalities in Reciprocal Social Interaction (QARSI), (2) Qualitative Abnormalities in Communication, (3) Restricted, Repetitive, and Stereotyped Patterns of Behavior, and (4) Abnormality of Development evident at or before 36 months.

Concerning the psychometric properties of the ADI-R, Bölte et al. (2006) report for the German version the following: regarding the interrater reliability for 27 of 36 algorithm-related items kappa values were *κ* > 0.70 (for the English original between *r* = 0.63 and *r* = 0.89 for the items of the diagnostic algorithm and *r* > 0.92 with regard to the scale scores of domains A–C). Retest reliabilities for the English version were between *r* = 0.93 and *r* = 0.97 for the scale scores of the domains.

#### Social Responsiveness Scale

The “Social Responsiveness Scale” (SRS, Bölte and Poustka, 2008) is a questionnaire of 65 items on social, communicative and rigid behavior in children and adolescents on a 4-point rating scale (1 = not true; 2 = sometimes true; 3 = often true; and 4 = almost always true). It is used for dimensional diagnostic and severity assessment of autism spectrum disorders or clarification of comorbid autistic traits in other clinical groups. It is completed by a caregiver of the respective child. Item 16, for example, addresses eye contact (“Avoids eye contact or has unusual eye contact.”).

Concerning the psychometric properties of the German version of the SRS, Bölte and Poustka (2008) report the following: retest reliabilities [norm sample with a time interval of 3 weeks–4 months: *r* = 0.80 for mother SRS (*N* = 107); *r* = 0.72 for father SRS (*N* = 76); mixed clinical sample for a time interval of 3–6 months: *r* = 0.95 (*N* = 49)] and internal consistencies (*α* = 0.93 for mother SRS, *α* = 0.91 for father SRS, and *α* = 0.97 for the mixed clinical sample). The convergent validity (examined on subsamples of the mixed clinical sample) with established instruments is mediocre: ADI-R (*N* = 113): subscale social interaction: *r* = 0.46; subscale communication: *r* = 0.40; subscale stereotypical behavior: *r* = 0.38; ADOS scale communication and social interaction (*N* = 119): *r* = 0.35.

#### IQ Assessment

As part of the diagnostic procedure for autism spectrum disorders, nearly all participants took an intelligence test. For two participants in the ASC group, externally assessed IQ scores were not available. Because both of them were attending regular schools without difficulties, we kept them in the study.

For the additional control group, the CBCL/4–18 (to exclude psychiatric comorbidity) and an intelligence test (CFT 20-R; in order to match with the clinical groups) were completed.

#### Facial Emotion Monitoring

The Facial Emotion Monitoring (FEMO) is an instrument developed in-house with the goal of rapidly surveying emotional behavior (facial expression and gesture) of participants by an independent rater during the ADOS diagnostic process. Relevant aspects are inquiries about social interaction and its quality, emotional expression, and psychomotor activity. The rating takes place within the standard situations specified by the items of the ADOS-2. The FEMO assessment sheet was compiled by an independent observer based on a video recording of the ADOS-2 to assess and rate facial and gestural expression, quality of social interaction, and psychomotility. Item 8a, for example, asks the observer to rate the assertion “The subject shows eye contact during the observation unit” on a 4-point rating scale.

### Procedure: Eye-Tracking During the ADOS-2 Session

The investigation with eye-tracking glasses took place in the framework of a regular ASC outpatient diagnostic procedure, using the gold standard diagnostic instruments apart from ADI-R (Bölte et al., 2006) and IQ assessment. Out of the clinic’s regular team for diagnostics of Autism Spectrum Disorder, 24 different ADOS interviewers (four male and 20 female) conducted the ADOS-2 in the study.

The Autism Diagnostic Observation Scale (Hus and Lord, 2014) serves as a basis for the acquisition of eye-tracking data. As the examined participants were exclusively children and adolescents from 8.0 to less than 18.0 years of age, with an IQ above 70, and with command of language as well as language fluency, only modules 3 and 4 were applied. For the acquisition of eye-gaze-behavior, we used the integrated interview activity *Conversation and Reporting* that is part of both modules. Following a short break, eye-tracking data were recorded during the second part of the ADOS-2 procedure. The participant put on the eye-tracking glasses; the examiner checked the correct position of the glasses and completed a three-point calibration ensuring valid recording of data before the interview began. Since accuracy is better if the calibration targets and the relevant stimuli are within the area encompassed by the calibration points (Holmqvist and Nyström, 2011), we defined the calibration points as a triangle around the visible region of the examiners body (head and upper part of the body).

In order to provide a framework for analysis, the first question in the interview section was defined as the beginning, while the participant’s last answer to the last question of the interview section was defined as the end of the sequence. Lengths of analyzed video segments varied between 5 min 38 s and 44 min 0 s (NTD: *M* = 923 s, *SD* = 241 s; non-ASC: *M* = 1,345 s, *SD* = 561 s; ASC: *M* = 1,345 s, *SD* = 561 s).

### Eye Movement Laboratory Procedures

Visual fixation patterns and dwell times were measured with eye-tracking equipment using hardware and software engineered by SMI (Teltow, Germany). The eye-tracking technology is video-based and uses dark-pupil/corneal reflection technique with eye-movement data collected at 60 Hz with binocular eye-tracking and integrated audio. The spatial resolution is 0.1°, and the gaze position accuracy is 0.5°. The eye-tracking glasses resemble ski-glasses, including an HD-Camera in the nose-bridge and binocular infrared sensors on the inside of the eye-glass frame. Thus, the HD-Camera records the visual field of the participant, while the binocular infrared-sensors gather data of the eye movements.

### Pre-processing of Gaze Data

#### Definition of Areas-of-Interest

In this study, we use percentages of total dwell time as the key measure to test our hypothesis. Total dwell time describes the cumulatively calculated duration of all fixations in relation to an AOI.

**Figure 2:**
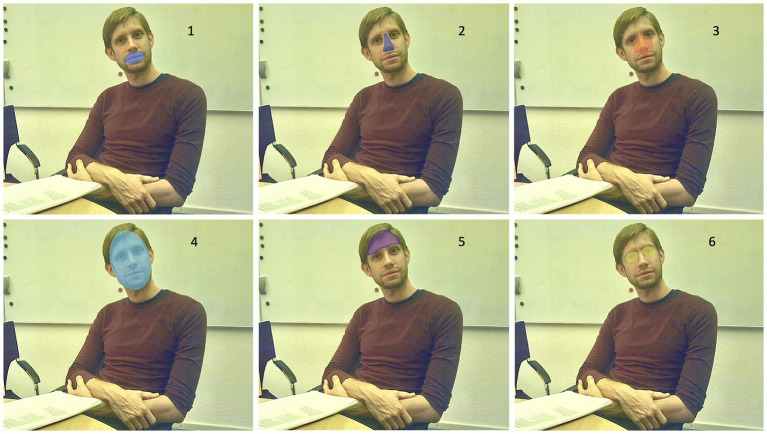
Illustrative example of the main AOIs: 1. Mouth, 2. Nose, 3. Center-Face, 4. Face, 5. Forehead, and 6. Eyes

For the empirical investigation of our hypothesis, we defined the following AOI: the eye region, including the left and the right eye of the interviewer not including the nasal root between them (EYES); the nose (NOSE); the mouth region of the interviewer (MOUTH); a circular area of interest in the middle of the face and a circle radius of 24 mm (referring to the face of a template, see section Fixation-based Semantic Gaze Mapping), comprising parts of the eye region and the nose (CENTER-FACE). Additional AOIs were the forehead (FOREHEAD), the chin (CHIN), and the entire face (FACE). Outside the face, we defined the following AOIs: the body of the interviewer without the face (BODY w/o HEAD) and the full body including the face (BODY WITH HEAD). The surrounding space outside the body of the interviewer is defined as white space (WHITESPACE). For an illustrative example of the template and its AOIs see Figure 2.

#### Fixation-Based Semantic Gaze Mapping

Pre-processing of raw eye-tracking data was performed with the BeGaze (version 3.7) analysis program by SMI (Teltow, Germany). The defined sequences of the interview were analyzed in a precise procedure. After a preliminary screening of the whole sequence to ensure data validity and exclude technical errors, the analysis of fixations and dwell times took place. Using a template with the defined AOIs, every fixation of the participant as recorded in the interview session was transferred manually to the corresponding region of the template face (a procedure called as “semantic gaze mapping”). The selected template is a representative front-shot of one of the diagnosticians who conducted the ADOS-2. Evaluation of the data was performed by blinded raters who had no information about group membership or diagnosis and had no knowledge about the coordinates or topography of the defined AOIs.

### Statistical Analysis

Because many AOI-related dwell times (and derived measures) are not stochastically independent from each other (some AOIs overlap or are even proper part of the other), no overall ANOVA with repeated measurements with AOI as dependent factor could be computed. To put special emphasis on differences between each clinical group (ASC or non-ASC) and the NTD control group, simple one-way ANOVAs between each clinical sample and the NTD group were conducted for each AOI. Effect sizes are reported in terms of standardized mean differences (SMD). Hedges’s *g*, rather than Cohen’s *d*, is used as an unbiased point estimator of effect sizes (Borenstein et al., 2009), because the former enables the computation of the 95% CI. These values are also the basis of the forest plot that provides a comprehensive review of the results.

Correlational analyses were conducted as follows: between AOI-based percentages of total dwell times with SRS scales, Pearson correlations were computed. Correlations with items concerning quality/frequency of eye contact in the SRS, ADOS-2, and FEMO instruments were performed by nonparametric Spearman rank-order correlations because of different scale properties of the items (ADOS-2 B1, for example, is dichotomous, whereas item 16 of the SRS is evaluated on a 4-point rating scale).

All statistical analyses are performed with SAS software, Version 9.4 (SAS Institute Inc., Cary, NC, USA). For hypothesis testing, a significance level of *α* = 0.05 was adopted.

## Results

### Sample Characteristics

Tables 1 and 2 summarize the characteristics for all three subsamples concerning quantitative and qualitative variables.

**Table 1:** Sample characteristics for quantitative variables of chronological age, IQ, and autistic symptomatology.

	NTD (*n* = 20)		Non-ASC (*n* = 26)		ASC (*n* = 19)	
	*M*	SD	*M*	SD	*M*	SD	*F*	*p*
Age	12.41	2.20	12.21	2.96	11.25	2.52	1.10	0.339
IQ^1^	109.80	9.48	99.46	17.47	107.29	15.69	3.04	0.055
SRS-T-Total	36.20	8.19	78.65	10.05	81.58	10.11	145.73	<0.0001
SRS-T-Awr	42.90	8.61	73.42	10.80	72.63	11.74	57.50	<0.0001
SRS-T-Cog	41.15	6.05	75.65	11.72	75.42	12.04	75.38	<0.0001
SRS-T-Com	40.00	6.88	81.35	12.74	87.16	12.51	107.42	<0.0001
SRS-T-Mot	41.05	7.36	73.00	10.39	80.00	11.26	89.81	<0.0001
SRS-T-RRB	47.45	4.76	74.96	7.82	76.37	9.85	91.71	<0.0001
ADI-R
QARSI			12.19	7.70	15.79	4.35	3.35	0.074
QAC			9.73	6.06	11.21	4.04	<1	
RRSPB			3.54	2.58	4.58	2.43	1.87	0.178
AbnDev			1.65	1.50	1.21	1.36	1.04	0.313

NTD, neurotypical development; ASC, autism spectrum condition; SRS, Social Responsiveness Scale; Awr, social awareness; Cog, social cognition; Com, social communication; Mot, social motivation; RRB, restricted interests and repetitive behavior; ADI-R, Autism Diagnostic Interview-Revised; QARSI, Qualitative Abnormalities in Reciprocal Social Interaction; QAC, Qualitative Abnormalities in Communication; RRSPB, Restricted, Repetitive, and Stereotyped Patterns of Behavior; AbnDev, Abnormality of Development evident at or before 36 months.

1 Two missing IQ values for two boys in the ASC group.

**Table 2:** Sample characteristics for the qualitative variables gender, main diagnoses, and co-morbid diagnoses.

	NTD (*n* = 20)		Non-ASC (*n* = 26)		ASC (*n* = 19)	
	*n*	%	*n*	%	*n*	%
Gender (f:m)	3:17	15.0:85.0	3:23	11.5:88.5	2:17	10.5:89.5
Main diagnosis	None		F90.[0;1]: 15 F43.2: 2 F32.2: 1 F81.2: 1 F92.0: 1 F92.8: 1 F93.2: 1 F94.0: 1 F98.8: 1 No Fxx-diag: 2		F84.0: 4 F84.1: 3 F84.5: 12	
Co-morbid diagnoses	None		Symptoms of AD(H)D or F90.0/F90.1: 1 F98.0: 3 F98.8: 3 F43.2: 2 F80.0: 2 F95.2: 1		Symptoms of AD(H)D or F90.0/F90.1: 8 F43.2: 2 F32.1: 1 F81.0: 1 F81.3: 1 F82: 1 F95.2: 1 Q86.0: 1	

F32.1, moderate depressive episode; F32.2, severe depressive episode without psychotic symptoms; F43.2, adjustment disorders; F80.0, specific speech articulation disorder; F81.0, specific reading disorder; F81.2, specific disorder of arithmetical skills; F81.3, mixed disorder of scholastic skills; F82, specific developmental disorder of motor function; F90.0, disturbance of activity and attention; F90.1, hyperkinetic conduct disorder; F92.0, depressive conduct disorder; F92.8, other mixed disorders of conduct and emotions; F93.2, social anxiety disorder of childhood; F94.0, elective mutism; F95.2, combined vocal and multiple motor tic disorder [de la Tourette]; F98.0, nonorganic enuresis; F98.8, other specified behavioral and emotional disorders with onset usually occurring in childhood and adolescence; Q86.0, fetal alcohol syndrome (dysmorphic).

There are no significant differences between the three groups with regard to chronological age. The same is true for IQ, although a trend can be seen [*F*(2, 60) = 3.04, *p* = 0.055] that is mainly caused by the lower mean in the non-ASC group (*M* = 99.46, *SD* = 17.47) as compared to the ASC and the NTD group (*M* = 107.29, *SD* = 15.69; *M* = 109.80, *SD* = 9.48, respectively). Regarding autistic symptomatology as assessed by the SRS, all six scales show significant differences between means (all *F*s > 57, all *p*s < 0.0001; see Table 1 for details). Gabriel’s *post-hoc* comparisons revealed that – in all cases – the means for the NTD group differed significantly from the means of the two clinical groups. Conversely, the means of the two clinical groups ASC and non-ASC did not differ significantly. Also, for the ADI-R, no significant differences between the two clinical groups could be noted; only a trend could be seen for the domain/scale QARSI, where the ASC group had more pronounced scores (*M* = 15.79, *SD* = 4.35 vs. *M* = 12.19, *SD* = 7.70 for non-ASC; *F*(1, 43) = 3.35, *p* = 0.074). Additionally, it can be noted that our ASC group seems to show less autistic symptomatology, because the scores were all lower than the one reported in the ADI-R manual by Rutter et al. (2003, Table 4, pp. 44–45): The corresponding values of their validation study are QARSI: *M* = 19.00, *SD* = 3.76; QAC: *M* = 16.33, *SD* = 2.96; RRSPB: *M* = 4.92, *SD* = 1.80.

Concerning the ADOS-2, it is not possible to report scale scores, since Module 3 and Module 4 have different items, different subscales, and different algorithms resulting in incommensurable scores. Therefore, we can just report the frequencies of the three ADOS-2 diagnoses “autism” (cutoffs: M3: 9; M4: 10), “autism spectrum” (cut-offs: M3: 7; M4: 7), and “non-spectrum” for the three groups: ASC: *n*(“autism”) = 9 (47.4%), *n*(“autism spectrum”) = 6 (31.6%), *n*(“non-spectrum”) = 4 (21.1%); non-ASC: *n*(“autism”) = 2 (7.7%), *n*(“autism spectrum”) = 6 (23.1%), *n*(“non-spectrum”) = 18 (69.2%); NTD: *n*(“autism”) = 0 (0.0%), *n*(“autism spectrum”) = 0 (0.0%), and *n*(“non-spectrum”) = 20 (100.0%). The frequencies of the three ADOS-2 diagnoses is significantly different between the three groups [χ^2^(4) = 30.41, *p* < 0.0001].

As can be seen in Table 2, the main ICD-10 diagnoses for the ASC group are childhood autism (F84.0: *n* = 4), atypical autism (F84.1: *n* = 3), and Asperger syndrome (F84.5: *n* = 12).

For the non-ASC group, various main diagnoses were obtained. The majority were diagnosed with hyperkinetic disorders (F90: *n* = 15), whereas other diagnoses were sparsely distributed. For six participants in the non-ASC group, the differential diagnosis of an autism spectrum disorder remained, but the diagnostic criteria had not been met at the time of the testing.

### AOI-Based Results

**Figure 3:**
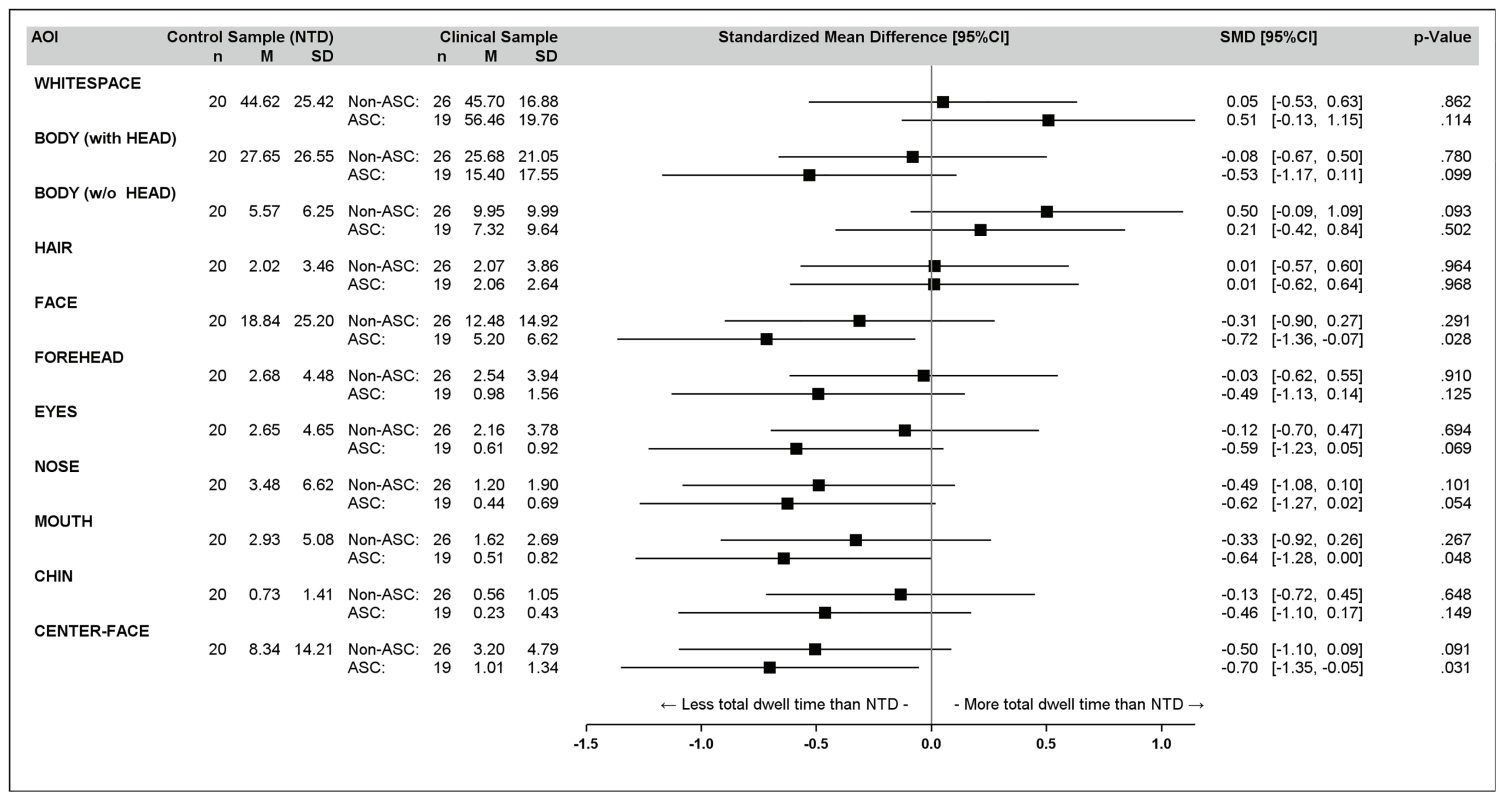
Forest plot of total dwell time percentages for different areas of interest (AOI).

In Figure 3, a forest plot of total dwell time percentages for different AOI is presented.

**Figure 4:**
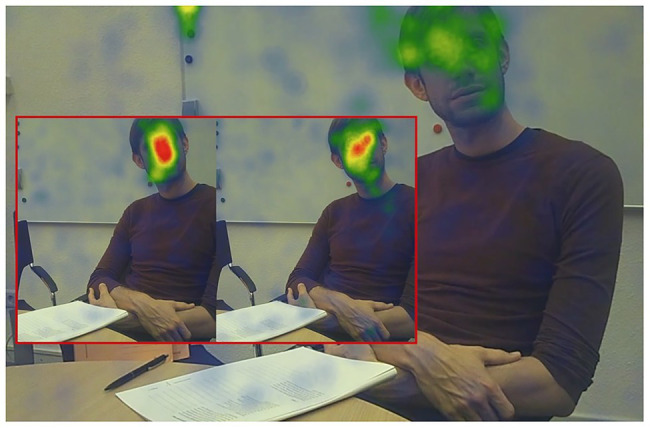
Heat map of fixations for the three groups (red box left side: NTD; red box right side: non-ASC; big picture: ASC) for the first 2 min of the ADOS‑2 “Conversation and Reporting” activity.

As can be seen in Figure 3, almost all descriptive statistics show that the ASC group differs more from the NTD group than the non-ASC group. For the AOIs CENTER-FACE and FACE, the NTDs show a significantly greater preference for the CENTER-FACE and the FACE than the ASCs [*F*(1, 37) = 5.00, *p* = 0.031, *g* = −0.70 and *F*(1, 37) = 5.22, *p* = 0.028, *g* = −0.72, respectively].

### Correlational Analyses

#### Correlations Between AOI-Based Measures and Degree of Autistic Symptomatology

Pearson correlations between AOI-based percentages of total dwell times with SRS scales are presented in Table 3. There were some significant correlations (BODY with HEAD – SRS-T-Awr, FACE – SRS-T-Mot, MOUTH – SRS-T-Mot, and MOUTH – SRS-T-Total), but the most and the highest correlations were obtained for the Nose and Central Face region.

**Table 3:** Intercorrelations of AOI-based measures and SRS scales.

	SRS Scale (T-scores)					
	Awr	Cog	Com	Mot	RRB	Total
**AOI**
WHITESPACE	0.131	0.092	0.091	0.192	0.082	0.115
BODY w/o HEAD	−0.075	−0.104	−0.052	−0.182	−0.085	−0.099
BODY WITH HEAD	**0.256** ^*^	0.232	0.201	0.109	0.189	0.211
HAIR	−0.064	−0.060	−0.023	−0.103	−0.101	−0.056
FACE	−0.207	−0.230	−0.160	**−0.253** ^*^	−0.175	−0.213
FOREHEAD	−0.103	−0.100	−0.073	−0.167	−0.083	−0.086
EYES	−0.139	−0.176	−0.083	−0.132	−0.076	−0.113
NOSE	**−0.254** ^*^	**−0.254** ^*^	−0.229	**−0.268** ^*^	−0.207	**−0.259** ^*^
MOUTH	−0.227	−0.217	−0.203	**−0.276** ^*^	−0.185	**−0.252** ^*^
CHIN	−0.049	−0.033	−0.060	−0.134	−0.096	−0.089
CENTER-FACE	**−0.269** *	**−0.278** ^*^	−0.240	**−0.284** ^*^	−0.220	**−0.273** ^*^

SRS, Social Responsiveness Scale; Awr, Social Awareness; Cog, Social Cognition; Com, Social Communication; Mot, Social Motivation; RRB, Restricted Interests and Repetitive Behavior.

* *p* < 0.05; significant correlations are shown in bold.

#### Correlations Between AOI-Based Measures and “Eye Contact”-Items From SRS, ADOS-2, and FEMO

In this section, the correlations of items concerning quality/frequency of eye contact in the SRS, ADOS-2, and FEMO instruments are presented. As a result of the different scale properties of the items (ADOS-2 B1, for example, is dichotomous, whereas item 16 of the SRS is evaluated on a 4-point rating scale), nonparametric Spearman rank-order correlations between percentages of total dwell times for the AOIs and these items were computed. All items are (re-)scaled in such a way that low values denote typical eye contact behavior, whereas higher values denote atypical eye contact. In summary, the AOI CenterFace belongs to the group with highest correlations with items concerning quality of eye contact (see Table 4).

**Table 4:** Spearman rank-order correlations of AOI-based measures items concerning quality of eye contact.

	SRS-I16	ADOS-2 B1	FEMO I8a
WHITESPACE	0.027	**0.468** ^***^	**0.421** ^***^
BODY w/o HEAD	0.038	**−0.293** ^*^	**−0.343** ^**^
BODY WITH HEAD	0.178	−0.151	−0.124
HAIR	−0.048	−0.168	−0.098
FACE	−0.031	**−0.319** ^**^	**−0.398** ^**^
FOREHEAD	0.009	−0.200	−0.209
EYES	−0.086	**−0.298** ^*^	**−0.367** ^**^
NOSE	−0.073	**−0.253** ^*^	**−0.445** ^***^
MOUTH	−0.036	−0.244	**−0.386** ^**^
CHIN	0.125	−0.141	−0.243
CENTER-FACE	−0.100	**−0.281** ^*^	**−0.421** ^***^

SRS-I16, Social Responsiveness Scale – Item 16 = avoiding eye-contact; unusual eye-contact; ADOS-2 B1, unusual eye-contact; FEMO I8a, FEMO Item 8a = eye-contact.

* *p* < 0.05;

** *p* < 0.01;

*** *p* < 0.001; significant correlations are shown in bold.

### Exploratory Results

Heat maps provide a quick and intuitive descriptive visual representation of eye-tracking data. They reveal the focus of visual attention and help to communicate important aspects of visual behavior.

In order to emphasize differences between all three groups visually, we created fixation-based heat maps for the first 2 min of the integrated interview activity “Conversation and Reporting.” As shown in Figure 4, the NTD group dwells in the central face area for the longest period; the non-ASC group also shows a predominant heat pattern in the center face area. Participants on tAS, however, show no identifiable focus or long-lasting dwell time for any relevant area that is associated with para-linguistic facial expression. Looking now at the distribution of dwell times in terms of the AOI FACE, the average dwell time of the NTD group is more than three times higher than that of the ASC group, and the circular area around the nasal root (CENTER FACE) exhibit the longest dwell times within the face.

## Discussion

The focal point in this study was the comparison of eye-gaze behavior in individuals on tAS vs. controls in an ecologically valid standard diagnostic situation corresponding to what Schilbach (2014) calls “online social cognition”. The results underline that the gaze behavior of individuals on tAS in an interactive interview situation with a real person differs from that of neurotypically developed controls. However, the differences do not seem to appear in the eye region, which is significantly less frequented by individuals on tAS in offline social cognition tasks. While there are indeed descriptive differences, statistically significant differences in eye-gaze behavior were confirmed by dwell times in the face, the mouth and in the central face region.

There is a large body of literature on the impact and relevance of direct eye contact for social cognition (Jones and Klin, 2013; Senju, 2013; Hietanen, 2018). Many authors suggest that direct gaze plays a dominant role in social communication (Csibra and Gergely, 2006) and that the white sclera – a unique characteristic that distinguishes human beings from other primate species – is an evolutionary development to improve the basic forms of human communication (Kobayashi and Kohshima, 1997; Farroni et al., 2004; Johnson, 2005). On the other hand, the eyes themselves are by no means an exclusive source for precise information about identity, emotional state, or mood of the observed person, quite apart from the fact that long-lasting eye contact elevates physiological arousal (Nichols and Champness, 1971) and provokes expectations of behavior.

One could thus presume that a balanced mix of mutual social interaction evinces a structured pattern of gaze sequences, which enables the interacting partners to read information efficiently from the face of the counterpart. In the field of reading research, there is evidence for preferred landing positions (PLP; Rayner, 1979) in sentence reading and of optimal viewing positions (OVP; O’Regan et al., 1984) in isolated word recognition (for a recent review, see Hyönä and Kaakinen, 2019). Given this background, the research efforts in object recognition identified similar PLPs and OVPs for optimal recognition performance (Foulsham and Kingstone, 2013).

The significant differences between NTD and ASC in our sample particularly in the AOI CENTER FACE reveal longer dwell times in the middle of the observed face in the NTD group. This arguably indicates that neurotypically developed face readers use this region as an optimal viewing position for successful categorization of facial expression.

In comparison with current studies it can be said that there are both representatives of an unimpaired holistic face categorization in ASC (Tanaka and Sung, 2016; Ventura et al., 2018) as well as researchers who assume an impairment in holistic face processing (Brewer et al., 2019). In an older offline study by Tanaka et al. (2012) it was found that individuals on tAS have a tendency to recognize the mouth region holistically, but the eyes as an isolated part of the face.

Apart from this many recent studies with an offline design suggest that emotion categorization is impaired in ASC (Uljarevic and Hamilton, 2012; Lozier et al., 2014; Velikonja et al., 2019). An eye-tracking study conducted in 2019 considered the question of how atypical face processing is related to differences in visual conjunctive processing (Stevenson et al., 2019). The study revealed that increasing ASC symptoms are associated with reduced levels of conjunctive processing. Although this offline study used photographs of virtual faces and the authors suggest untypical visual conjunctive processing in ASC, there are no indications for a starting point of conjunctive face processing in ASC.

Notably, Hsiao and Cottrell (2008) found that an optimal position for face recognition is around the center of the nose. However, this is contrary to the results of a large number of lab studies, which indicate that the eyes and the mouth region are highly relevant for face recognition. This suggests that there are differences in PLP and OVP between offline lab studies and real online social interaction (Foulsham, 2020). One reason for this may be that the online character of ecologically valid social situations has other prerequisites than an offline experiment with a precisely defined task. Constructive and active participation in a real-time social interaction is associated with a different approach to cognitive processing that is characterized by reciprocal relations as opposed to situations in which social phenomena are merely observed (De Jaegher, 2008; Schilbach, 2010; Wilms et al., 2010).

The reciprocity of social interactions demands an implicit repertoire of rapid and flexible processes in a circular operational sequence of action and reaction. Whereas offline social cognition is only based on an observer position without the additional cognitive load of being involved in an interaction, the participant in a socially interactive process is only able to react adequately if the constantly flowing information can be categorized in the context of the developing situation and in compliance with his own social schemas (Schaller and Rauh, 2017; Schaller, 2019). In order to make efficient use of the face of the counterpart, one must possess implicit face-detection strategies, capturing all relevant hints for a better understanding of the social situation.

Looking now at the visual scan pathways of the three groups, it can be ascertained that the NTD group in particular shows significantly longer dwell times for the circular area around the nasal root (CENTER FACE). This is astonishing, because a direct gaze in the eyes of the counterpart occurs less frequently than on the forehead or the mouth.

It follows, therefore, that with regard to the distribution of the AOIs in the face, the main focus is not in the eyes. Instead, there is evidence that the region around the nose is the most visited and revisited area of interest in the face. NTD tend to dwell eight times longer in the center face area than the ASC group. This phenomenon can be visually presented by comparing the heat maps of both groups. While the NTD group develops a clear center face preference in the heat map within a timeframe of less than 2 min (see Figure 4), the distribution of fixations in the ASC group shows an unstructured spread of seemingly uncoordinated scanpaths without a clear focus on any of the relevant AOI for facial information.

The majority of fixations in the ASC group lie outside the face or in parts that do not provide any information about facially expressed emotions (hair, ears, and chin). Based on this result, we suggest that neurotypically developed individuals have an implicit automatism, using the center of the face as the Archimedean Point from which the facial expression can be gathered as a valid source of information. Furthermore, our results are supported by Bobak et al. (2017), who showed that the dwell times on the eye region did not correlate with face perception skills of controls, while there was a significant and robust correlation between the ability to recognize faces and dwell time spent on the nose.

A further indication for the tendency to use the center of the face as optimal viewing position for a better recognition and categorization of facial expression can be found in so called “Super Recognisers,” who outperform neurotypical individuals in face recognition (Russell et al., 2009). Individuals who meet the criteria for super recognition use the nose instead of the eyes to achieve an efficient distribution of spatial attention across the face, resulting in higher-than-average face recognition (Bobak et al., 2017).

The significant differences between groups concerning the mouth region are consistent with the findings in offline social cognition that individuals on tAS spend less time on the mouth region as compared to their neurotypically developing peers (Wagner et al., 2013).

Turning to an analysis of correlations between AOI-based measures and the degree of autistic symptomatology, the AOIs NOSE and CENTER FACE reveal the highest correlations with regard to social responsiveness, in so far that high rates in the SRS total score result in shorter dwell times for CENTER-FACE.

Looking particularly at the AOI CENTER-FACE, we find the highest correlations with the SRS subscales Social Cognition and Social Motivation. The subscale Social Cognition is defined as the ability to adequately interpret social key stimuli, while the subscale Social Motivation reflects the need for social interaction. It is, therefore, a fair assumption that the use of the center face as an ideal basis for implicit face-detection strategies is a relevant criterion for social interaction abilities.

Furthermore, the correlation between AOI based measures and the ADOS-2/B1 item “unusual eye-contact” initially shows an expected pattern of significant positive correlation concerning WHITESPACE and a significant negative correlation with respect to EYES. However, there are also significant correlations for the AOIs NOSE and CENTER-FACE. The highest correlation can be found for FACE and ADOS-2 B1, which suggests that, from the rater’s perspective, a participant’s glance in the face of the diagnostician can be a sufficient indicator for neurotypical eye-contact. On the other hand, it raises the question as to whether the rater is able to differentiate between actual mutual gaze and a fixation of the nasal root or one of the eyebrows.

In order to have a third-party assessment of the ADOS-2 “Conversation and Reporting” activity in terms of emotional behaviors, such as facial and gestural expression, quality of social interaction, and psychomotility, we compiled the FEMO. Initially, it was used to prove the extent to which the ADOS rater assessment corresponds with the FEMO observation and the eye-tracking data. In this context, item 8a is particularly important, with high values denoting unusual and minimal eye-contact. Here, we also find the highest significant negative correlations in the AOIs NOSE and CENTER FACE and a highly significant positive correlation for the AOI WHITESPACE.

Thus, it can be concluded that gaze behavior in an ecologically valid online social situation clearly differs from offline situations.

### Limitations

The limitations of the study are, first, that current results are based on a relatively small sample. Therefore, only large effects could be detected. In order to generate more conclusive data that can detect small and medium effect sizes, it would be appropriate to develop study concepts involving large numbers of participants.

Concerning the statistical analyses, the multiple comparisons’ problem arose (1) in analyses concerning group differences for the various AOIs and (2) in the correlational analyses. Because of lack of stochastic independence for the total dwell time-related measures in (1), no justifiable adjustments for alpha could be made. For the correlational analyses, we ran the analyses without alpha adjustments, since there is no gold standard how to deal with the multiple comparisons’ problem – a problem that is still under debate (e.g., Rubin, 2017), and for which Bonferroni correction seems to be a suboptimal solution having its own problems (e.g., inflation of type II errors; see Perneger, 1998). Therefore, the question of whether our results are reliable should be answered by replication studies.

Thirdly, we only tested children and adolescents from 8 to less than 18 years of age. A wider spectrum of age ranges, including younger children, adults, and older participants, could offer further information about the development of gaze behavior during online social situations in participants with and without ASC.

Furthermore, this sample had a negligible proportion of female participants, so that no gender-specific differences could be evaluated for. Future investigation of gaze behavior may help to find gender specific differences.

In this study, we chose to focus on the gaze behavior of the ASC group. The non-ASC group turned out to be a very heterogeneous cohort, with too many different diagnoses to run additional analyses.

Moreover, we only investigated individuals on tAS without intellectual disabilities, which makes it impossible to generalize the results for all individuals on tAS. With regard to autistic symptoms, our group is more likely to show less pronounced severity. Thus, the extent of untypical gaze-behavior in our ASC group may underestimate the real extent of deviating gaze-behavior in people with ASC who do not have co-occurring intellectual disabilities.

Lastly, we used eye-tracking data without any other psychophysiological parameters. Future research in online social cognition might combine eye-tracking and psychophysiological measures in order to clarify any existing correlations.

### Conclusion

With the face being a projection surface for expression, its interpretation is dependent on the spectrum of a performer’s facial expressions and the repertoire of emotional expressive categories and social schemas available to the observer. The central face seems to be the hot spot, where many socially relevant behavioral expressions as well as social information perceptual processes meet.

Additionally, contextual factors, like underlining gestures, body movements, paraverbal signs, and sceneries, specifically influence the perception and categorization of facial stimuli (Aviezer et al., 2017). Thus, it is important to keep track of the counterpart’s face while considering contextual variables or the general setting of a certain social situation (Pfeiffer et al., 2013).

The results of this study show that it is not the eyes but the central face region that is an important anchor point in using all the above-mentioned factors efficiently.

While in neurotypical individuals this implicit and procedural development takes place in an emergent process of exchange with the social environment, it seems that this development is different in individuals on tAS.

Consequently, it will be necessary to analyze this process in further studies of online social interaction, particularly by comparing factors such as contextual background and social schemas. In parallel, a clinical study with enlarged number of participants and a broader age range, considering children, adolescents and adults is under way.

#### Data Availability Statement

The datasets presented in this article are not readily available because of confidentiality reasons. Requests to access the datasets should be directed to reinhold.rauh@uniklinik-freiburg.de.

## Ethics Statement

The studies involving human participants were reviewed and approved by the Ethics Committee of the University of Freiburg. Written informed consent to participate in this study was provided by the participants’ legal guardian/next of kin. Written informed consent was obtained from the individual(s) for the publication of any potentially identifiable images or data included in this article.

## Author Contributions

US, RR, and CF developed the study concept and designed the study with assistance of AB, AI, and MH. AB, AI, and MH coordinated the study, recruited the participants, and completed data collection. AB and AI checked the integrity and the accuracy of the gaze data and performed the gaze data pre-processing pipeline with supervision of US and RR. RR planned and carried out the statistical analyses with contributions of US and MH. All authors made contributions to the interpretation of the data. US, RR, and AB drafted the initial manuscript with contributions of AI, CF, MB, MH, and LT. All authors reviewed and revised the manuscript and approved the submission of the final manuscript.

### Conflict of Interest

The authors declare that the research was conducted in the absence of any commercial or financial relationships that could be construed as a potential conflict of interest.

## References

[ref1] Augoustinos M. Walker I. (1995). “Social schemas” in Social cognition. eds. Augoustinos M. Walker I. (London: SAGE Publications), 346.

[ref2] Aviezer H. Ensenberg N. Hassin R. R. Gomes De Mesquita B. Feldman Barrett L. (2017). The inherently contextualized nature of facial emotion perception. Curr. Opin. Psychol. 17, 47–54. 10.1016/j.copsyc.2017.06.006, PMID: 28950972

[ref3] Baron-Cohen S. Wheelwright S. (2004). The empathy quotient: an investigation of adults with asperger syndrome or high functioning autism, and normal sex differences. J. Autism Dev. Disord. 34, 163–175. 10.1023/B:JADD.0000022607.19833.00, PMID: 15162935

[ref4] Bartlett F. C. (1932). Remembering: A study in experimental and social psychology. London: Cambridge University Press.

[ref5] Bobak A. K. Parris B. A. Gregory N. J. Bennetts R. J. Bate S. (2017). Eye-movement strategies in developmental prosopagnosia and “super” face recognition. Q. J. Exp. Psychol. 70, 201–217. 10.1080/17470218.2016.1161059, PMID: 26933872

[ref65] Bölte S. Poustka F. (2004). Diagnostische beobachtungsskala für autistische störungen (ADOS): erste ergebnisse zur zuverlässigkeit und gültigkeit. Z. Kinder. Jugendpsychiatr. Psychother. 32, 45–50. 10.1024/1422-4917.32.1.45 14992047

[ref6] Bölte S. Poustka F. (2008). SRS: skala zur erfassung sozialer reaktivität. dimensionale autismus-diagnostik [German version of Social Responsiveness Scale (SRS) by John N. Constantino and Christian P. Gruber]. Bern: Huber.

[ref7] Bölte S. Rühl D. Schmötzer G. Poustka F. (2006). Diagnostisches Interview für Autistische Störungen revidiert (ADI-R). Deutsche Fassung des Autism Diagnostic Interview-revised. Bern: Huber.

[ref62] Borenstein M. Hedges L. V. Higgins J. P. T. Rothstein H. R. (2009). Introduction to meta-analysis. Chichester: Wiley.

[ref8] Brewer R. Bird G. Gray K. L. H. Cook R. (2019). Face perception in autism spectrum disorder: modulation of holistic processing by facial emotion. Cognition 193. 10.1016/j.cognition.2019.104016, PMID: 31280061

[ref10] Chevallier C. Parish-Morris J. Mcvey A. Rump K. M. Sasson N. J. Herrington J. D.. (2015). Measuring social attention and motivation in autism spectrum disorder using eye-tracking: stimulus type matters. Autism Res. 8, 620–628. 10.1002/aur.1479, PMID: 26069030 PMC4624010

[ref11] Chita-Tegmark M. (2016). Social attention in ASD: a review and meta-analysis of eye-tracking studies. Res. Dev. Disabil. 8, 620–628. 10.1016/j.ridd.2015.10.011, PMID: 26547134

[ref12] Csibra G. Gergely G. (2006). “Social learning and social cognition: the case for pedagogy” in Processes of change in brain and cognitive development. Attention and performance XXI. eds. Munakata Y. Johnson M. H. (Oxford: Oxford University Press), 249–274.

[ref13] Dalton A. N. Chartrand T. L. Finkel E. J. (2010). The Schema-Driven Chameleon: how mimicry affects executive and self-regulatory resources. J. Pers. Soc. Psychol. 98, 605–617. 10.1037/a0017629, PMID: 20307132

[ref14] De Jaegher H. (2008). Social understanding through direct perception? Yes, by interacting. Conscious. Cogn. 18, 535–542. 10.1016/j.concog.2008.10.007 19091603

[ref15] Farroni T. Johnson M. H. Csibra G. (2004). Mechanisms of eye gaze perception during infancy. J. Cogn. Neurosci. 16, 1320–1326. 10.1162/0898929042304787, PMID: 15509381

[ref16] Faul F. Erdfelder E. Lang A.-G. Buchner A. (2007). G*Power 3: a flexible statistical power analysis program for the social, behavioral, and biomedical sciences. Behav. Res. Methods 39, 175–191. 10.3758/BF03193146 17695343

[ref17] Foulsham T. (2020). “Beyond the picture frame: the function of fixations in interactive tasks” in Psychology of learning and motivation – Advances in research and theory. Vol. 73, eds. Federmeier K. D. Schotter E. R. (Cambridge, MA: Academic Press Inc.), 33–58.

[ref18] Foulsham T. Kingstone A. (2013). Optimal and preferred eye landing positions in objects and scenes. Q. J. Exp. Psychol. 66, 1707–1728. 10.1080/17470218.2012.762798, PMID: 23398283

[ref19] Frazier T. W. Strauss M. Zetzer E. E. Hardan A. Y. Eng C. Youngstrom E. A. (2017). A meta-analysis of gaze differences to social and nonsocial information between individuals with and without Autism HHS Public Access. J. Am. Acad. Child Adolesc. Psychiatry 56, 546–555. 10.1016/j.jaac.2017.05.005, PMID: 28647006 PMC5578719

[ref20] Greenbaum P. E. Dedrick R. F. Lipien L. (2004). “The child behavior checklist/4-18(CBCL/4-18)” in Comprehensive handbook of psychological assessment. Vol. 2. eds. Hersen M. Hilsenroth M. J. Segal D. L. (Hoboken, NJ: John Wiley & Sons), 179–181.

[ref60] Guillon Q. Hadjikhani N. Baduel S. Rogé B. (2014). Visual social attention in autism spectrum disorder: insights from eye tracking studies. Neurosci. Biobehav. Rev. 42, 279–297. 10.1016/j.neubiorev.2014.03.013 24694721

[ref22] Hietanen J. K. (2018). Affective eye contact: an integrative review. Front. Psychol. 9:1587. 10.3389/fpsyg.2018.01587, PMID: 30210410 PMC6121038

[ref23] Holmqvist K. Nyström M. (2011). Eye-tracking—A comprehensive guide to methods and measures. New York: Oxford University Press.

[ref24] Hsiao J. H. W. Cottrell G. (2008). Two fixations suffice in face recognition. Psychol. Sci. 19, 998–1006. 10.1111/j.1467-9280.2008.02191.x, PMID: 19000210 PMC7360057

[ref25] Hus V. Lord C. (2014). The autism diagnostic observation schedule, module 4: revised algorithm and standardized severity scores. J. Autism Dev. Disord. 44, 1996–2012. 10.1007/s10803-014-2080-3, PMID: 24590409 PMC4104252

[ref26] Hyönä J. Kaakinen J. K. (2019). “Eye movements during reading” in Eye movement research: An introduction to its scientific foundations and applications. eds. Klein C. Ettinger U. (Cham, CH: Springer), 239–274.

[ref27] Jack R. E. Schyns P. G. (2015). The human face as a dynamic tool for social communication. Curr. Biol. 25, R621–R634. 10.1016/j.cub.2015.05.052, PMID: 26196493

[ref28] Johnson M. H. (2005). Subcortical face processing. Nat. Rev. Neurosci. 6, 766–774. 10.1038/nrn1766, PMID: 16276354

[ref29] Jones W. Carr K. Klin A. (2008). Absence of preferential looking to the eyes of approaching adults predicts level of social disability in 2-year-old toddlers with autism spectrum disorder. Arch. Gen. Psychiatry 65, 946–954. 10.1001/archpsyc.65.8.946, PMID: 18678799

[ref30] Jones W. Klin A. (2013). Attention to eyes is present but in decline in 2-6-month-old infants later diagnosed with autism. Nature 504, 427–431. 10.1038/nature12715, PMID: 24196715 PMC4035120

[ref31] Klin A. Jones W. Schultz R. Volkmar F. Cohen D. (2002). Visual fixation patterns during viewing of naturalistic social situations as predictors of social competence in individuals with autism. Arch. Gen. Psychiatry 59, 809–816. 10.1001/archpsyc.59.9.809, PMID: 12215080

[ref32] Kobayashi H. Kohshima S. (1997). Unique morphology of the human eye. Nature 387, 767–768. 10.1038/42842, PMID: 9194557

[ref33] Kuzmanovic B. Georgescu A. L. Eickhoff S. B. Shah N. J. Bente G. Fink G. R.. (2009). Duration matters: dissociating neural correlates of detection and evaluation of social gaze. NeuroImage 46, 1154–1163. 10.1016/j.neuroimage.2009.03.037, PMID: 19328236

[ref34] Lakin J. L. Jefferis V. E. Cheng C. M. Chartrand T. L. (2003). The chameleon effect as social glue: evidence for the evolutionary significance of nonconscious mimicry. J. Nonverbal Behav. 27, 145–161. 10.1023/A:1025389814290

[ref35] Lozier L. M. Vanmeter J. W. Marsh A. A. (2014). Impairments in facial affect recognition associated with autism spectrum disorders: a meta-analysis. Dev. Psychopathol. 26, 933–945. 10.1017/S0954579414000479, PMID: 24915526

[ref36] Luhmann N. (1987). Soziale Systeme. Suhrkamp Verlag: Frankfurt am Main.

[ref37] Nichols K. A. Champness B. G. (1971). Eye gaze and the GSR. J. Exp. Soc. Psychol. 7, 623–626. 10.1016/0022-1031(71)90024-2

[ref39] O’Regan J. K. Lévy-Schoen A. Pynte J. Brugaillère B. (1984). Convenient fixation location within isolated words of different length and structure. J. Exp. Psychol. Hum. Percept. Perform. 10, 250–257. 10.1037/0096-1523.10.2.250, PMID: 6232343

[ref40] Papagiannopoulou E. A. Chitty K. M. Hermens D. F. Hickie I. B. Lagopoulos J. (2014). A systematic review and meta-analysis of eye-tracking studies in children with autism spectrum disorders. Soc. Neurosci. 9, 610–632. 10.1080/17470919.2014.934966, PMID: 24988218

[ref41] Perneger T. V. (1998). What’s wrong with Bonferroni adjustments. Br. Med. J. 316, 1236–1238. 10.1136/bmj.316.7139.1236, PMID: 9553006 PMC1112991

[ref42] Pfeiffer U. J. Timmermans B. Vogeley K. Frith C. D. Schilbach L. (2013). Towards a neuroscience of social interaction. Front. Hum. Neurosci. 7:22. 10.3389/fnhum.2013.00022, PMID: 23378836 PMC3561599

[ref43] Rayner K. (1979). Eye guidance in reading: fixation locations within words. Perception 8, 21–30. 10.1068/p080021, PMID: 432075

[ref44] Rubin M. (2017). Do *p* values lose their meaning in exploratory analyses? It depends how you define the familywise error rate. Rev. Gen. Psychol. 21, 269–275. 10.1037/gpr0000123

[ref45] Russell R. Duchaine B. Nakayama K. (2009). Super-recognizers: people with extraordinary face recognition ability. Psychon. Bull. Rev. 16, 252–257. 10.3758/PBR.16.2.252, PMID: 19293090 PMC3904192

[ref67] Rutter M. Le Couteur A. Lord C. (2003). ADI-R: The autism diagnostic interview-revised. Los Angeles, CA: Western Psychological Services.

[ref46] Schaller U. M. (2019). Autism and Schema. Complex Social Cognition and Social Schemas in Autism Spectrum Disorders. Albert-Ludwigs-Universität Freiburg im Breisgau.

[ref68] Schaller U. M. Biscaldi M. Fangmeier T. Tebartz van Elst L. Rauh R. (2019). Intuitive moral reasoning in high-functioning autism spectrum disorder: a matter of social schemas? J. Autism Dev. Disord. 49, 1807–1824. 10.1007/s10803-018-03869-y 30610668

[ref47] Schaller U. M. Rauh R. (2017). What difference does it make? Implicit, explicit and complex social cognition in autism spectrum disorders. J. Autism Dev. Disord. 47, 961–979. 10.1007/s10803-016-3008-x, PMID: 28083780

[ref48] Schank R. C. Abelson R. P. (1977). Scripts, plans, goals and understanding: An inquiry into human knowledge structures. Hillsdale: Erlbaum.

[ref49] Schilbach L. (2010). A second-person approach to other minds. Nat. Rev. Neurosci. 11:449. 10.1038/nrn2805-c1, PMID: 20485366

[ref50] Schilbach L. (2014). On the relationship of online and offline social cognition. Front. Hum. Neurosci. 8, 1–8. 10.3389/fnhum.2014.00278, PMID: 24834045 PMC4018539

[ref51] Senju A. (2013). Atypical development of spontaneous social cognition in autism spectrum disorders. Brain Dev. 35, 96–101. 10.1016/j.braindev.2012.08.002, PMID: 22964276

[ref52] Simon H. A. (1956). Rational choice and the structure of the environment. Psychol. Rev. 63, 129–138. 10.1037/h0042769, PMID: 13310708

[ref53] Stevenson R. A. Philipp-Muller A. Hazlett N. Wang Z. Y. Luk J. Lee J.. (2019). Conjunctive visual processing appears abnormal in autism. Front. Psychol. 9:2668. 10.3389/fpsyg.2018.02668, PMID: 30713514 PMC6346680

[ref54] Tanaka J. W. Sung A. (2016). The “Eye Avoidance” hypothesis of autism face processing. J. Autism Dev. Disord. 46, 1538–1552. 10.1007/s10803-013-1976-7, PMID: 24150885 PMC3997654

[ref69] Tanaka J. W. Wolf J. M. Klaiman C. Koenig K. Cockburn J. Herlihy L.. (2012). The perception and identification of facial emotions in individuals with autism spectrum disorders using the Let’s Face It! Emotion Skills Battery. J. Child Psychol. Psychiatr. 53, 1259–1267. 10.1111/j.1469-7610.2012.02571.x PMC3505257 22780332

[ref55] Uljarevic M. Hamilton A. (2012). Recognition of emotions in autism: a formal meta-analysis. J. Autism Dev. Disord. 43, 1517–1526. 10.1007/s10803-012-1695-5, PMID: 23114566

[ref56] Velikonja T. Fett A. -K. Velthorst E. (2019). Patterns of nonsocial and social cognitive functioning in adults with autism spectrum disorder a systematic review and meta-analysis. JAMA Psychiat. 76, 135–151. 10.1001/jamapsychiatry.2018.3645, PMID: 30601878 PMC6439743

[ref57] Ventura P. Carmo J. C. Souza C. Martins F. Leite I. Pinho S.. (2018). Holistic processing of faces is intact in adults with autism spectrum disorder. Vis. Cogn. 26, 13–24. 10.1080/13506285.2017.1370051

[ref58] Wagner J. B. Hirsch S. B. Vogel-Farley V. K. Redcay E. Nelson C. A. (2013). Eye-tracking, autonomic, and electrophysiological correlates of emotional face processing in adolescents with autism spectrum disorder. J. Autism Dev. Disord. 43, 188–199. 10.1007/s10803-012-1565-1, PMID: 22684525 PMC3913826

[ref59] Wilms M. Schilbach L. Pfeiffer U. Bente G. Fink G. R. Vogeley K. (2010). It’s in your eyes—using gaze-contingent stimuli to create truly interactive paradigms for social cognitive and affective neuroscience. Soc. Cogn. Affect. Neurosci. 5, 98–107. 10.1093/scan/nsq024, PMID: 20223797 PMC2840847

